# Polo-Like Kinase 1 (PLK1) Is Involved in Toll-like Receptor (TLR)-Mediated TNF-α Production in Monocytic THP-1 Cells 

**DOI:** 10.1371/journal.pone.0078832

**Published:** 2013-10-18

**Authors:** Jinyue Hu, Guihua Wang, Xueting Liu, Lina Zhou, Manli Jiang, Li Yang

**Affiliations:** 1 Medical Research Center, Changsha Central Hospital, Changsha, Hunan, China; 2 Cancer Center, Changsha Central Hospital, Changsha, Hunan, China; 3 Central Laboratory, Renmin Hospital of Wuhan University, Wuhan, Hubei, China; 4 Tuberculosis Research Center, Changsha Central Hospital, Changsha, Hunan, China; INRS - Institut Armand Frappier, Canada

## Abstract

Polo-like kinases (PLKs) have been reported to be essential components of anti-viral pathways. However, the role of PLKs in the production of pro-inflammatory cytokines induced by TLR activation is uncertain. We report here that monocytic THP-1 cells expressed PLK1, PLK2, PLK3 and PLK4. When THP-1 cells were treated with GW843682X, an inhibitor of PLK1 and PLK3, the results showed that GW843682X down-regulated Pam3CSK4- and LPS-induced TNF-α at both the gene and protein levels. GW843682X did not impact Pam3CSK4-induced IL-1β and IL-8 or LPS-induced IL-1β, but it down-regulated LPS-induced IL-8 significantly. Moreover, western blot results showed that TLRs activated PLK1, and PLK1 inhibition by RNA interference down-regulated Pam3CSK4-induced TNF-α production, suggesting the involvement of PLK1 in TNF-α up-regulation. In addition, GW843682X treatment for 12 to 24 h induced cell death and down-regulated MyD88, but neither of these roles contributed to the down-regulation of TNF-α, as TNF-α gene expression was up-regulated at 1 h. Furthermore, GW843682X inhibited Pam3CSK4-induced activation of ERK and NF-κB, which contributed to Pam3CSK4-induced up-regulation of TNF-α. GW843682X also inhibited LPS-induced activation of ERK, p38 and NF-κB, which contributed to LPS-induced up-regulation of TNF-α. Taken together, these results suggested that PLK1 is involved in TLR2- and TLR4-induced inflammation, and GW843682X may be valuable for the regulation of the inflammatory response.

## Introduction

Toll-like receptors (TLRs) are type I transmembrane proteins, and they function as pattern recognition receptors (PRRs) to detect the conserved structures of pathogens known as pathogen-associated molecular patterns (PAMPs) [1,2]. TLRs play important roles in the induction of the innate immune response to form the first line of the host to defense the infection of the pathogens [1,2], and they are essential for efficient activation of adaptive immunity [2-4]. The activation of TLRs elicits multiple signaling events, leading to the induction of pro-inflammatory cytokines and resulting in the activation of the innate immune response. The dimerization of all human TLRs except TLR3 recruits TIR domain-containing adaptor proteins, including TIR-associated protein (TIRAP) and myeloid differentiation factor 88 (MyD88), which associates with IL-1R-associated kinases (IRAKs) and TNFR-associated factor 6 (TRAF6), resulting in the activation of nuclear factor (NF)-κB and the production of pro-inflammatory cytokines [5]. Meanwhile, the dimerization of TLR3 and TLR4 recruits the TIR-domain-containing adaptor protein-inducing IFN-β (TRIF), which associates with TRAF3, leading to the activation of NF-κB and interferon regulatory factor 3 (IRF3) and the production of type I interferons [5].. 

TLR activation induces multiple pathways that mediate pro-inflammatory responses. In addition to the canonical TLR-MyD88 and TLR-TRIF signals, other signaling pathways have been reported to be involved in TLR signal transduction, and they function to regulate the TLR-mediated immune response. Glycogen synthase kinase 3, which is a kinase that regulates the body’s metabolism through the phosphorylation of glycogen synthase and other substrates [6], has been reported to regulate TLR-mediated production of pro- and anti-inflammatory cytokines [7,8]. The inhibition of glycogen synthase kinase 3 signaling shifted the TLR-mediated pro-inflammatory response to the anti-inflammatory response via activation of the CREB pathway [7,8]. ABCA1-activated protein kinase 4 has been reported to promote IL-10 production induced by TLR4 [9]. By inhibition of ERK-mediated NF-κB activation, Notch signaling decreases TLR-triggered inflammatory responses in macrophages [10]. In addition, protein kinases D1 and D2 have been reported to be involved in chemokine production induced by TLR2, 4, and 5 [11].

Polo-like kinases (PLKs) function to regulate mitosis, and they play important roles in the maintenance of genomic stability [12]. Recently, PLK2 and PLK4 have been reported to be essential components of antiviral pathways in vitro and in vivo, and they activate a signaling branch involving a dozen proteins [13]. However, the roles of PLKs in the production of pro-inflammatory cytokines induced by TLR activation are uncertain. In our study, we found that the activation of TLRs induced the phosphorylation of PLK1. The inhibition of PLK1 down-regulated Pam3CSK4- and LPS-induced TNF-α production, suggesting that PLK1 signaling was involved in the TLR-induced inflammatory response.

## Materials and Methods

### Cell lines and reagents

The monocytic THP-1 cell line was purchased from the American Type Culture Collection (ATCC, Manassas, VA). Cells were grown in RPMI 1640 medium containing 10% FCS, 100 units/ml penicillin, and 100 mg/ml streptomycin. 

Rabbit anti-human phosphorylated PLK1, ERK, JNK, p38, and NF-κB p65 antibodies, rabbit anti-human PLK1, PLK3, PLK4, NF-κB p65, and β-actin antibodies, and the ERK inhibitor U0126 were purchased from Cell Signaling Technology (Beverly, MA, USA). Human PLK interference RNA, rabbit anti-human PLK2 (Snk, sc-25421), and rabbit anti-human MyD88, were purchased from Santa Cruz Biotechnology (Santa Cruz, CA, USA). The cytokine TNF-α, IL-1β, and IL-8 ELISA kits and the fluorescein isothiocyanate (FITC)-annexin V/propidium iodide (PI) apoptosis kits were purchased from Jiamay Biotech. (Beijing, China). The PLK1 inhibitor GW843682X (5-(5,6-Dimethoxy-1*H*- benzimidazol-1-yl)-3-[[2- (trifluoromethyl)phenyl]methoxy]-2-thiophenecarboxamide) was purchased from Tocris (Ellisville, MO, USA). The JNK inhibitor SP600125, the p38 inhibitor SB203580, and the NF-κB inhibitor BAY 11-7082 were purchased from Enzo Life Sciences (San Diego, CA). The TLR1/2 ligand Pam3CSK4 was purchased from InvivoGen (San Diego, CA). The TLR4 ligand lipopolysacchride (LPS) was purchased from Sigma-Aldrich (St. Louis, MO). The mouse anti-human TLR2 and TLR4 mAbs were purchased from R&D systems (Minneapolis, MN).

### Preparation of Macrophages

Female BALB/c mice (6 - 8 weeks old) were purchased from the Shanghai Laboratory Animal Center (Shanghai, China). Thioglycollate-elicited macrophages were obtained from mice that were injected intraperitoneally with 1 ml of 3.8% thioglycollate broth (Sigma-Aldrich). Macrophages were harvested 4 days later by peritoneal lavage. The cells were cultured in RPMI 1640 medium supplemented with 10% heat-inactivated FCS and antibiotics at 37 °C in an atmosphere of humidified 5% CO_2_. After incubation for 1 h, the non-adherent cells were removed and the adherent cells were cultured for the subsequent studies.

### Reverse transcription-PCR (RT-PCR)

Total RNA was extracted from 1 to 2 × 10^6^ cells using TRIzol (Invitrogen, Carlsbad, CA, USA), as described by the manufacturer. mRNA was reverse transcribed with RevertAid (MBI Fermentas, Burlington Ontario, Canada) at 42 °C for 60 min, and the resulting cDNA was subjected to PCR (94 °C for 1 min followed by 20-25 cycles at 94 °C for 30 s, 60 °C for 30 s, and 68 °C for 1 min and an extension for 10 min at 68 °C). The PCR products were separated on 1.0% agarose gels and visualized with ethidium bromide. The forward and reverse primer pairs are listed (5΄ to 3΄) as follows:

β-actin-F, TCGTGCGTGACATTAAGGAGA,
β-actin-R, ATACTCCTGCTTGCTGATCCA;
GAPDH-F, AATCCCATCACCATCTTCCA,
GAPDH-R, CCTGCTTCACCACCTTCTTG;
IL-1β-F, TGAACTGAAAGCTCTCCACCT,
IL-1β-R, ACTGGGCAGACTCAAATTCCA;
IL8-F, TTGGCAGCCTTCCTGATTT,
IL8-R, TCAAAAACTTCTCCACAACCC;
MyD88-F, ACTTGGAGATCCGGCAACT,
MyD88-R, TGGAAGTCACATTCCTTGCT;
PLK1-F, CCCCTCACAGTCCTCAATAA,
PLK1-R, TGTCCGAATAGTCCACCC;
PLK2-F, TTCGGGGATGTCTGGAAAAC,
PLK2-R, ATCTCCACCATCCATGAGGTT;
PLK3-F, ATGAGGTCTCCGGTTTGGTGA,
PLK3-R, AGTTGATACCCAAAGCCGAA;
PLK4-F, CACTCAGCAGAAATGCTTTCA,
PLK4-R, TGTCCTTCTGCAAATCTGGA;
TLR2-F, AAGGGAATTGGTTGCAGGAT,
TLR2-R, ACAGATTACAGTTGGCCCTCT;
TLR4-F, CAGCAGGAACACTTACCTGGA,
TLR4-R, TTTATTCCCTCTGCACTGGA;
TNFα-F, ATCAGAGGGCCTGTACCTCAT,
TNFα-R, AGACTCGGCAAAGTCGAGATA;

### Immunoblot

Cells (1 - 2 × 10^6^) were lysed in 200 ml lysis buffer (20 mM Tris, pH 7.5, 150 mM NaCl, 1% Triton X-100, 1 mM EDTA, 1 mM sodium pyrophosphate, 1 mM β-glycerophosphate, 1 mM Na_3_VO_4_, 1 mg/ml leupeptin). The cell lysate was centrifuged at 12,000 g at 4 °C for 5 min. Nuclear protein extraction was performed using a nuclear and cytoplasmic protein extraction kit (Beyotime, Haimen, China) according to the manufacturer's instructions. Proteins were electrophoresed on 10% SDS-PAGE gels, and transferred onto Immobilon P membranes (Millipore, Billerica, MA, USA). The membranes were blocked by incubation in 3% nonfat dry milk for 1 h at room temperature and then incubated with primary antibodies in PBS containing 0.01% Tween 20 overnight at 4 °C. After incubation with a horseradish peroxidase-conjugated secondary antibody, the protein bands were detected with SuperSignal chemiluminescent substrate-stable peroxide solution (Pierce Rockford, IL, USA) and BIOMAX-MR film (Eastman Kodak Co., Rochester, NY, USA). When necessary, the membranes were stripped with Restore Western blot stripping buffer (Pierce) and re-probed with antibodies against various cellular proteins.

### Quantitative real time RT-PCR (qRT-PCR)

The qRT-PCR was performed as described by Sun et al. [14]. Briefly, total RNA from the cells was isolated and reverse transcribed as described above. The cDNA was amplified using TaqMan Universal PCR master mix (Applied Biosystems, Foster City, CA, USA) and an ABI Prism 7500 sequence detection system (Applied Biosystems). The amplification of the target genes was normalized using the amplification levels of glyceraldehyde-3-phosphate dehydrogenase (GAPDH) as an endogenous control. The efficiency of the PCR was tested by amplification of the target from serially diluted cDNA generated from the reverse transcription of a stock set of human RNA. The data analysis and calculations were performed using the 2^−ΔΔCT^ comparative method, as described by the manufacturer. Gene expression is shown as the fold induction of a gene measured in TLR ligand-treated samples relative to samples cultured with medium.

### Flow cytometric analysis

Cell death was detected by fluorescein isothiocyanate (FITC)-annexin V/propidium iodide (PI) staining as described by Xiao et al. [15]. Briefly, 1 - 2 × 10^6^ cells were washed twice with PBS and then labeled with FITC-annexin V and PI in binding buffer according to manufacturer’s instructions. The fluorescence signals were detected on a FACScan (BD Bioscience, San Jose, CA). The log of FITC-annexin V–fluorescence was displayed on the x-axis, and the log of PI fluorescence was displayed on the y-axis. For each analysis, 10,000 events were recorded. 

For TLR2 and TLR4 protein detection, THP-1 cells, cultured for 24 h in 6-well plates, were harvested and washed with fluorescence-activated cell sorting (FACS) buffer (5 mM EDTA, 0.1% NaN3, and 1% FCS, in Dulbecco’s phosphate-buffered saline (PBS)). After incubation with a monoclonal antibody against human TLR2 or TLR4 for 40 min on ice, the cells were stained with a FITC-labeled secondary antibody and examined for protein expression by flow cytometry (BD Biosciences).

### siRNA transfection

siRNA against human PLK1 (sc-36277) and a silencer negative siRNA control (sc-37007) were purchased from Santa Cruz Biotechnology (Santa Cruz, CA, USA). siRNA transfection reagent (sc-29528, Santa Cruz, CA, USA) was used to transfect siRNA into THP-1 cells according to the manufacturer's instructions. Briefly, 0.5 μg siRNA and 6 µl siRNA transfection reagent were used for each transfection (0.5 × 10^6^ cells/well in 6-well plates). The cells were cultured with siRNA for 5 h, then washed with medium, and cultured with fresh medium for 48 h. The expression of PLK1 was analyzed by qRT-PCR and western blot**.**

### Enzyme-linked immunosorbent assay

The production of IL-1β, TNF-α and IL-8 in culture supernatants was detected by enzyme-linked immunosorbent assay (ELISA) according to the manufacturers’ standard protocols.

### Statistical analysis

All experiments were performed at least three times, and the representative results are shown. The results are expressed as the mean ± S.D. Differences between groups were examined for statistical significance using Student’s *t* test, and *P* values equal to or less than 0.05 were considered statistically significant ( n = 3 for each qRT-PCR and ELISA test).

## Results

### The expression of PLKs in THP-1 cells

**Figure 1:**
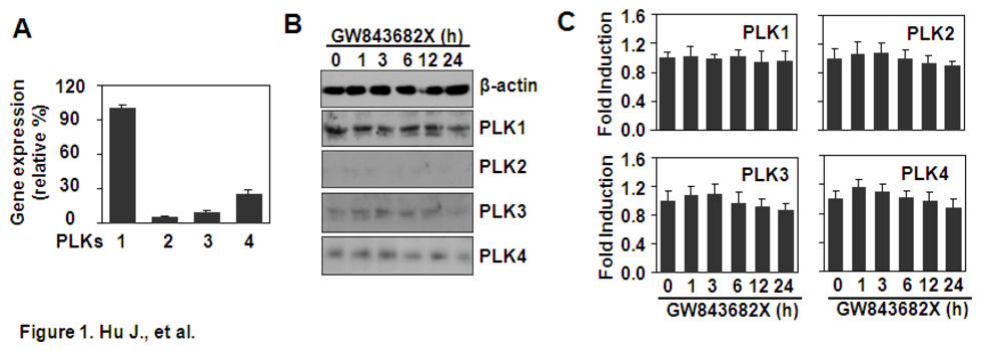
The expression of PLKs in THP-1 cells. (A) Quantitative expression of the *PLK* genes. THP-1 cells were cultured for 24 h in 6-well plates. The gene expression of *PLKs* was detected by qRT-PCR. *PLK1* gene expression was designated as 100%. *PLK2-4* gene expression was shown as percentage expression compared with *PLK1* *gene* *expression*. (B) The effect of GW843682X on the protein expression of the PLKs. THP-1 cells were treated with 10 µM GW843682X for the indicated time periods. The protein expression of PLKs was detected by western blot. β-actin protein was detected as a loading control. (C) The effect of GW843682X on the gene expression of the *PLKs*. THP-1 cells were treated with 10 µM GW843682X for the indicated time periods. The gene expression of the *PLKs* was detected by qRT-PCR. Gene expression in the GW843682X-treated groups was shown as the fold expression relative to the non-treated groups.

We first detected the gene expression of *PLKs* in THP-1 cells. The qRT-PCR results showed that THP-1 cells expressed high levels of *PLK1*, low levels of *PLK2* and *PLK3*, and moderate levels of *PLK4* (Figure 1A). Subsequently, the western blot results showed that the rest THP-1 cells expressed high levels of the PLK1 protein, low levels of the PLK2 and PLK3 proteins, and moderate levels of PLK4 protein (Figure 1B). When THP-1 cells were treated with GW843682X, an inhibitor of PLK1 and PLK3, the qRT-PCR results showed that GW843682X did not affect the gene expression of *PLK1, PLK2, PLK3*, or *PLK4* (Figure 1C). Western blot results showed that GW843682X did not affect the protein expression of PLK1, PLK2, PLK3, or PLK4 (Figure 1B), suggesting that GW843682X did not affect the expression of PLKs.

### GW843682X inhibits the expression of cytokines induced by Pam3CSK4 and LPS

**Figure 2:**
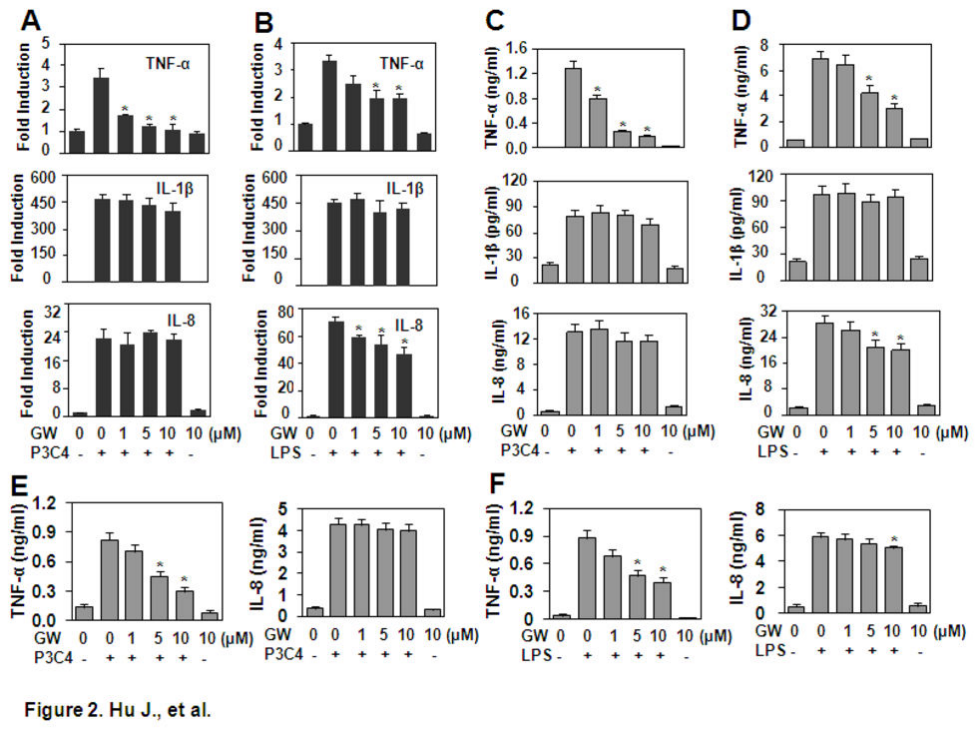
The effect of GW843682 (GW) on cytokine expression induced by Pam3CSK4 and LPS. (A, B) Gene expression in THP-1 cells. The cells, pre-treated with the indicated concentrations of GW for 30 min, were re-stimulated with 1 µg/ml Pam3CSK4 (P3C4) (A) or 1 µg/ml LPS (B) for 1 h. The gene expression of *TNF-α*, *IL-1β* and *IL-8* was detected by qRT-PCR. (C, D) Protein expression in THP-1. The cells pre-treated with the indicated concentrations of GW for 30 min were re-stimulated with 1 µg/ml Pam3CSK4 (P3C4) (C) or LPS (D) for 24 h. The cytokine proteins secreted in the supernatant were detected by ELISA. (E, F) The protein expression of cytokines in primary mouse peritoneal macrophages. Mouse peritoneal macrophages were prepared as described in Materials and Methods and treated as described in (C) (E) or (D) (F). The cytokine proteins secreted in the supernatant were detected by ELISA. * *P* < 0.05 compared with the Pam3CSK4- or LPS-treated groups in (A-F).

PLK2 and PLK4 have been reported to be involved in the regulation of anti-viral pathways in vivo and in vitro [13]. In this study, the effect of PLK1 and PLK3 inhibition on the TLR2- and TLR4-induced inflammatory response was detected in monocytic THP-1 cells. The qRT-PCR and ELISA results showed that GW843682X down-regulated the expression of TNF-α induced by Pam3CSK4 or LPS and the expression of IL-8 induced by LPS at both the gene and protein levels (Figures 2A-D), but it did not affect the expression of IL-1β induced by Pam3CSK4 or LPS, or the expression of IL-8 induced by Pam3CSK4 (Figures 2A-D). Moreover, we detected the effect of GW843682X on the cytokine production induced by LPS and Pam3CSK4 in primary mouse peritoneal macrophages. The ELISA results showed that GW843682X down-regulated the expression of TNF-α induced by LPS or Pam3CSK4 (Figures 2E-F). Only 10 μM GW843682X slightly down-regulated the expression of IL-8 induced by LPS (Figure 2F). Moreover, GW843682X did not affect expression of IL-8 induced by Pam3CSK4 (Figure 2E).

### PLK1 RNA interference down-Regulated Pam3CSK4-Induced TNF-α

**Figure 3:**
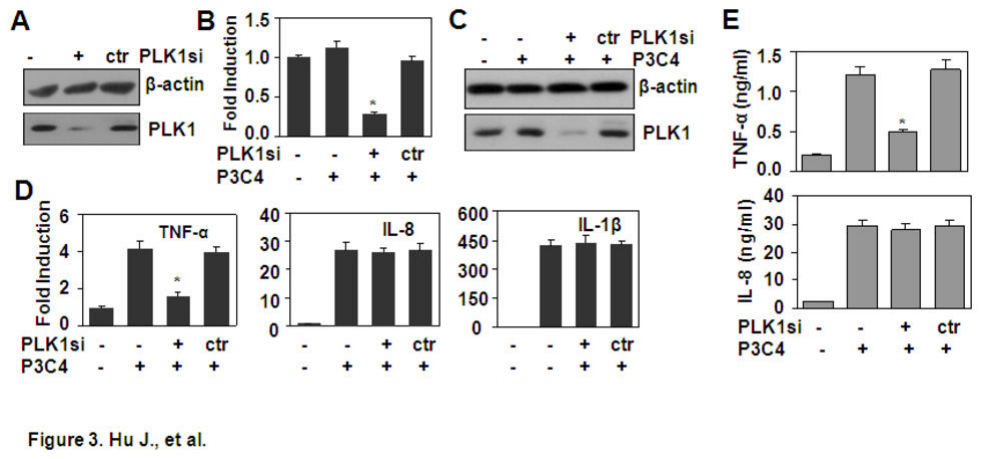
The effect of PLK1 RNA interference on Pam3CSK4-induced cytokine production. (A-C) The effect of PLK1 siRNA on PLK1 expression. THP-1 cells, transfected with PLK1 siRNA as described in Materials and Methods, were untreated (A) or stimulated with 1 µg/ml Pam3CSK4 (P3C4) for 1 h (B-C). The protein expression of PLK1 was detected by western blot (A, C). The gene expression of PLK1 was detected by qRT-PCR (B). * *P* < 0.05 compared with the non-treated groups. (D) PLK1 RNA interference down-regulated cytokine transcript expression. THP-1 cells, transfected with PLK1 siRNA, were stimulated with 1 µg/ml Pam3CSK4 (P3C4) for 1 h. The transcript expression of *TNF-α*, *IL-1β*, and *IL-8* were detected by qRT-PCR. * *P* < 0.05 compared with the non-treated group. (E) The effect of PLK1 siRNA on cytokine protein expression. THP-1 cells, transfected with PLK1 siRNA, were stimulated with 1 µg/ml Pam3CSK4 (P3C4) for 24 h. The protein expression of TNF-α and IL-8 was detected by ELISA. * *P* < 0.05 compared with the Pam3CSK4-treated alone group.

GW843682X is an inhibitor of PLK1 and PLK3. To confirm whether high expression of PLK1 is involved in TLR-mediated TNF-α production, the expression of PLK1 was inhibited by RNA interference. Western blot results showed that PLK1 siRNA inhibited the protein expression of PLK1 (Figure 3A,C). The qRT-PCR results showed that PLK1 siRNA inhibited the gene expression of *PLK1* (Figure 3B). Meanwhile, PLK1 siRNA down-regulated the gene expression of *TNF-α* induced by Pam3CSK4, but it did not regulate the gene expression of *IL-1β* or the gene expression of *IL-8* induced by Pam3CSK4 (Figure 3D). Moreover, PLK1 siRNA down-regulated the protein expression of TNF-α induced by Pam3CSK4, but it did not regulate the protein expression of IL-8 (Figure 3E). These results suggest that PLK1 is involved in the production of TNF-α.

### The effect of Pam3CSK4 and LPS on phosphorylation of PLK1 and the gene expression of PLKs

**Figure 4:**
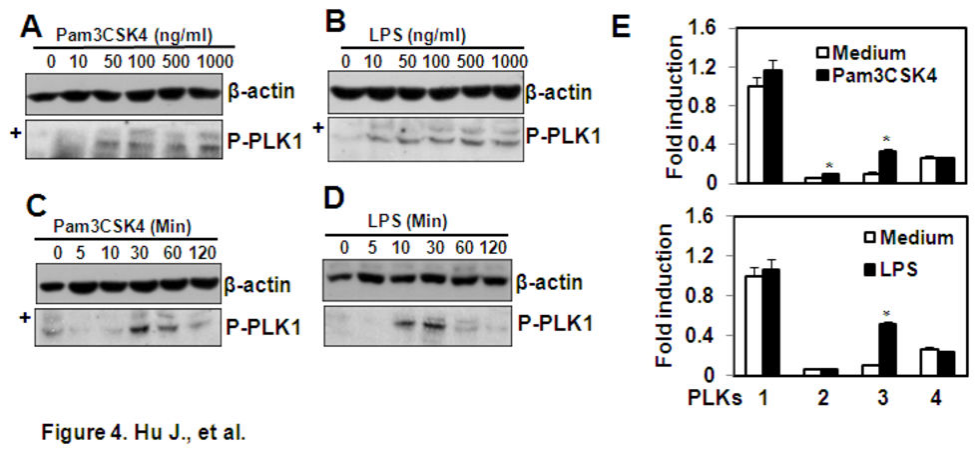
The effect of Pam3CSK4 and LPS on PLK activation and expression . (A, B) Pam3CSK4 and LPS induce dose-dependent activation of PLK1. THP-1 cells were treated with the indicated concentrations of Pam3CSK4 (A) or LPS (B) for 30 min. The phosphorylation of PLK1 was detected by western blot. β-actin protein was detected as loading control. ‘+’ indicated the non-specific bands. (C, D) Pam3CSK4 and LPS induced time-dependent activation of PLK1. The THP-1 cells were treated with 1 µg/ml Pam3CSK4 (C) or LPS (D) for the indicated time periods. The phosphorylation of PLK1 was detected by western blot. β-actin protein was detected as loading control. ‘+’ indicated the non-specific bands. (E) The effect of Pam3CSK4 and LPS on PLK gene expression. THP-1 cells were treated with 1 µg/ml Pam3CSK4 or LPS for 24 h. The gene expression of *PLKs* was detected by qRT-PCR. The *PLK1* gene expression in non-treated group was designated as 1-fold. The *PLK1* gene expression in Pam3CSK4- or LPS-treated groups, and the *PLK2-4* gene expression in all groups was shown relative to the *PLK1* gene expression in the non-treated group. * *P* < 0.05 compared with the control groups respectively.

To further demonstrate the involvement of PLK1 in Pam3CSK4- and LPS-induced TNF-α expression, the effect of Pam3CSK4 and LPS on the phosphorylation of PLK1 was detected by western blot. The results showed that both Pam3CSK4 and LPS induced the phosphorylation of PLK1 in a dose- and time-dependent manner (Figures 4A-D). In addition, Pam3CSK4 treatment up-regulated the gene expression of *PLK2* and *PLK3*, and LPS treatment up-regulated the gene expression of *PLK3* (Figure 4E). These results suggested that the TLR signaling affected the expression and function of PLKs.

### GW843682X induces cell apoptosis in THP-1 cells

**Figure 5:**
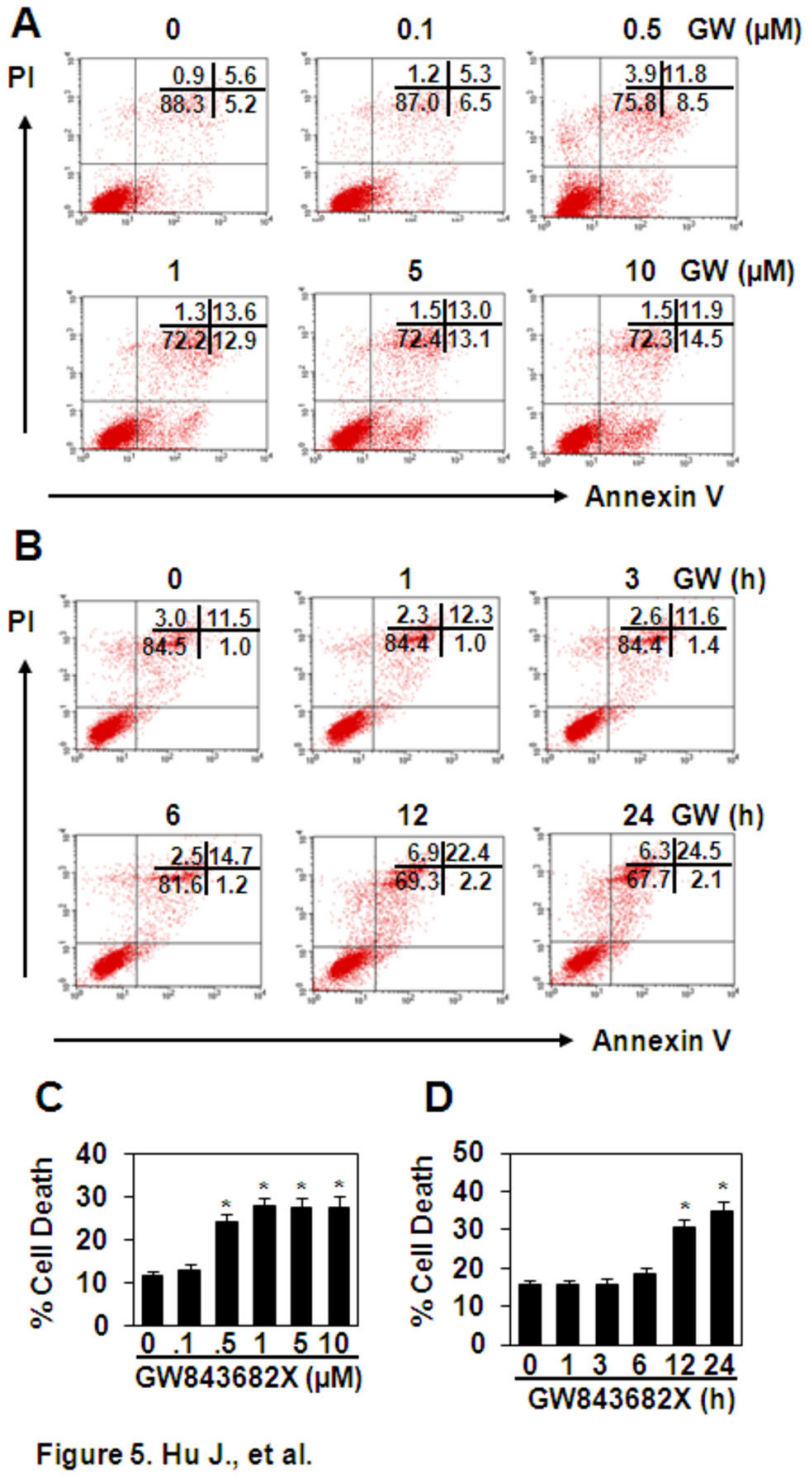
The effect of GW843682X (GW) on THP-1 apoptosis. (A) GW induced dose-dependent cell death. THP-1 cells were treated with the indicated concentrations of GW for 24 h. Unfixed cells were stained with FITC-annexinV/PI. Cell apoptosis was measured by flow cytometry. (B) GW induced time-dependent cell death. THP-1 cells were treated with 10 μM GW for the indicated time periods. Cell apoptosis was measured as described in (A). (C) The quantitative data for (A). (D) The quantitative data for (B). * *P* < 0.05 compared with the non-treated groups in (C) and (D).

PLKs have been reported to regulate cell proliferation through their roles in the entry into mitosis, centrosome maturation, bipolar spindle formation, segregation of the chromosomes, cytokinesis, and monitoring the fidelity of the checkpoint control itself [16]. The inhibition of PLK1 results in cell apoptosis [17-20], and it has been validated as a target for cancer therapy. To test whether the down-regulation of TNF-α induced by Pam3CSK4 or LPS is related to the GW843682-induced cell apoptosis, THP-1 cells were treated with GW843682X at various concentrations for various time periods, and cell apoptosis was detected by FITC-Annexin V/PI staining. The results showed that GW843682X induced cell apoptosis at dosages from 0.5 to 10 μM and with treatment times ranging from 12 to 24 h (Figures 5A-D). As Pam3CSK4 and LPS induced the up-regulation of cytokine gene expression at 1 h, and GW843682X specifically down-regulated Pam3CSK4-induced TNF-α, LPS-induced TNF-α and IL-8, we concluded that cell apoptosis was not responsible for the down-regulation of the Pam3CSK4- or LPS-induced cytokines.

### The effect of GW843682X on the expression of TLR2, TLR4, and MyD88

**Figure 6:**
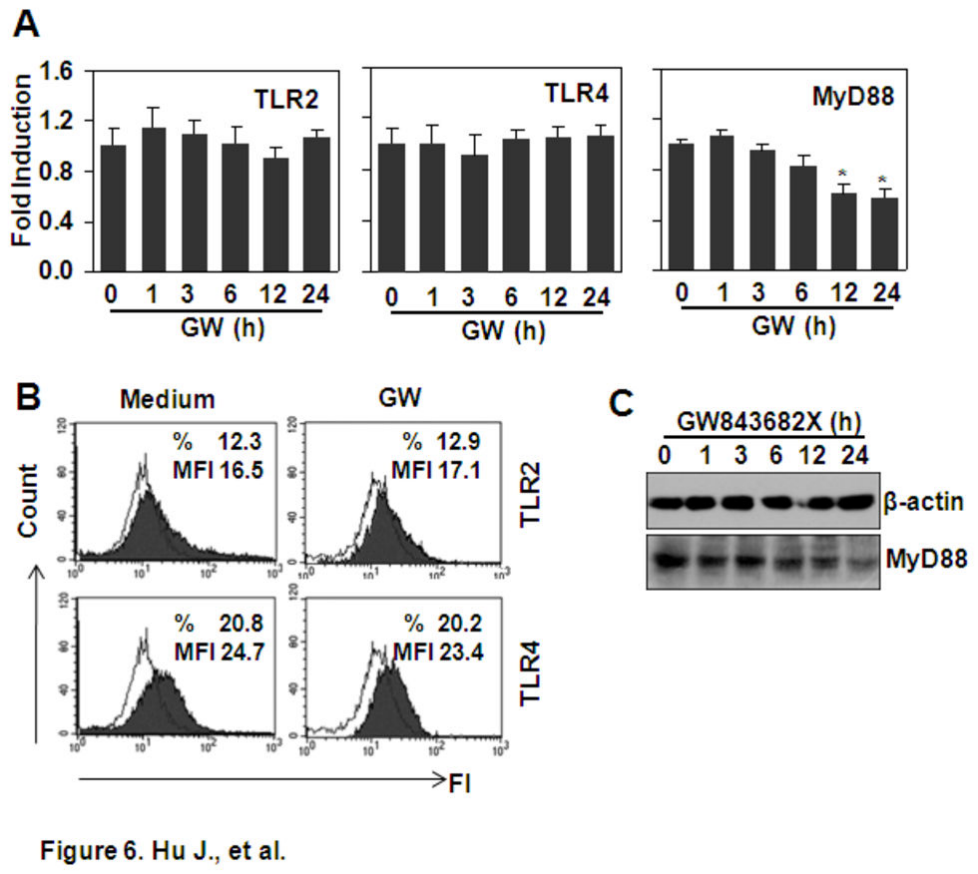
The effect of GW843682X (GW) on the expression of TLR2, TLR4, and MyD88. (A) The effect of GW on the gene expression of *TLR2, TLR4*, and *MyD88*. THP-1 cells were treated with 10 µM GW for the indicated time periods. The gene expression of *TLR2*, *TLR4*, and *MyD88* was detected by qRT-PCR. * *P* < 0.05 compared with the non-treated group. (B) The effect of GW on the protein expression of TLR2 and TLR4. THP-1 cells were treated with 10 µM GW for 24 h. TLR2 and TLR4 protein expression was detected by FACS. (C) The effect of GW on the protein expression of MyD88. THP-1 cells were treated with 10 µM GW for the indicated time periods. The protein expression of MyD88 was detected by western blot. β-actin protein expression was detected as loading control.

The down-regulation of a receptor or its signal adaptor protein contributes to the decrease of the ligand-induced response. To test the effect of GW843682X on the expression of TLR2, TLR4, and their signal adaptor MyD88, THP-1 cells were treated with 10 μM GW843682X for 0 to 24 h, and the gene expression of *TLR2*, *TLR4*, and *MyD88* was detected by RT-PCR. The results showed that GW843682X treatment did not affect the expression of *TLR2* and *TLR4* (Figure 6A), but it down-regulated the expression of *MyD88* from 12 to 24 h (Figure 6A). Correspondingly, the FACS results showed that GW843682X treatment for 24 h did not regulate the protein expression of TLR2 and TLR4 (Figure 6B), and the western blot results showed that GW843682X treatment down-regulated the protein expression of MyD88 in a time-dependent manner (Figure 6C). However, the down-regulation of MyD88 did not contribute to the down-regulation of Pam3CSK4- or LPS-induced cytokines for the reason mentioned above.

### GW843682X down-regulates TNF-α expression via the inhibition of MAPK and NF-κB signaling

**Figure 7:**
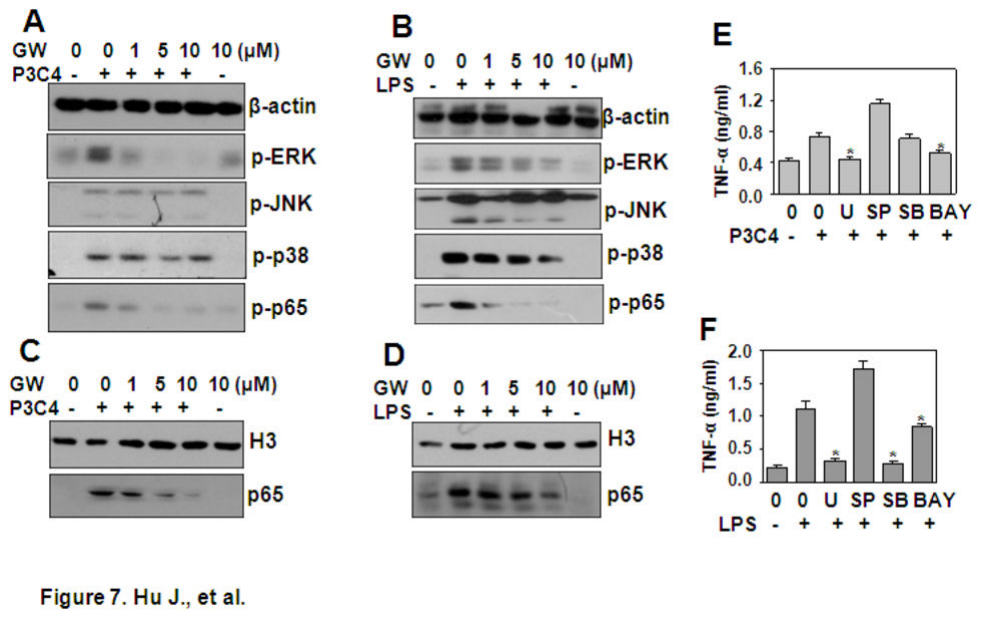
GW843682X (GW) down-regulates TNF-α expression via inhibition of MAPK and NF-κB signaling. (A, B) The effect of GW on Pam3CSK4- and LPS-induced signal transduction. THP-1 cells, starved overnight and pre-treated with the indicated concentrations of GW for 30 min, were re-stimulated with 1 µg/ml Pam3CSK4 (P3C4) (A) or LPS (B) for 30 min. The phosphorylation of ERK, JNK, p38, and NF-κB p65 was detected by western blot. β-actin protein expression was detected as loading control. (C, D) The effect of GW on Pam3CSK4- and LPS-induced NF-κB p65 nuclear localization. THP-1 cells were treated as in (A) (C) and (B) (D). Total proteins in the nucleus were extracted, and NF-κB p65 protein was detected by western blot. Histone H3 protein expression was detected as a loading control. (E, F) Pam3CSK4 and LPS induced TNF-α via the MAPK and NF-κB pathways. THP-1 cells, pre-treated with 10 µM U0126 (U), or SP600125 (SP), or SB203580 (SB), or Bay11-3072 (BAY) for 30 min, were re-stimulated with 1 µg/ml Pam3CSK4 (P3C4) (E) or LPS (F) for 24 h. TNF-α secreted in the supernatant was detected by ELISA. * *P* < 0.05 compared with the Pam3CSK4- or LPS-treated groups.

TLRs activate the MAPK and NF-κB signaling pathways to induce the pro-inflammatory response. To test whether GW843682X down-regulated Pam3CSK4- and LPS-induced TNF-α by regulation of MAPKs and NF-κB, THP1 cells that had been pre-treated with various concentrations of GW843682X for 30 min were re-stimulated with 1 µg/ml Pam3CSK4 or LPS. The phosphorylation of MAPKs, including ERK, JNK, and p38, and the phosphorylation of NF-κB p65 was detected by western blot. The results showed that GW843682X pre-treatment dose-dependently down-regulated Pam3CSK4-induced activation of ERK and p65 (Figure 7A), which contributed to the induction of TNF-α (Figure 7E). Meanwhile, GW843682X pre-treatment down-regulated LPS-induced activation of ERK, p38, and p65 dose-dependently (Figure 7B), which contributed to the induction of TNF-α (Figure 7F). GW843682X inhibition of NF-κB signaling was also detected by measurement of the nuclear localization of NF-κB p65 total protein induced by TLR activation (Figures 7C-D). Taken together, we concluded that the inhibition of PLK1 down-regulated TLR2- and TLR4-induced TNF-α via inhibition of MAPK and NF-κB signaling.

## Discussion

In this study, we found that Pam3CSK4 and LPS induced the phosphorylation of PLK1. The inhibition of PLK1 down-regulated Pam3CSK4- and LPS-induced TNF-α, which was due to the inhibition of MAPK and NF-κB signal transduction, suggesting that PLK1 was involved in the production of TNF-α induced by TLR activation. 

The PLKs function to regulate mitosis in a wide range of cell types [21]. PLK1 has been reported to be over-expressed in numerous cancer types, such as non-small-cell lung cancer, oropharyngeal carcinoma, esophageal carcinoma, melanoma, colorectal cancer, hepatoblastoma, non-Hodgkin lymphoma, and others [12]. The over-expression of PLKs often correlates with a poor patient prognosis [22]. PLKs have also been reported to be expressed in dendritic cells and to be involved in the induction of the anti-virus immune response [13]. In this study, we found that THP-1 cells expressed high levels of PLK1, low levels of PLK2 and PLK3, and moderate level of PLK4. Therefore, we focused our study on PLK1, and found that PLK1 was activated by treatment with the TLR ligands, Pam3CSK4 and LPS. Meanwhile, inhibition of PLK1 with the inhibitor GW843682X down-regulated TNF-α production induced by Pam3CSK4 and LPS. These results suggested that PLK1 was involved in the induction of TNF-α. 

Consistent with the reported observation that the inhibition of PLKs induces cell apoptosis [17,18,20], we found that the treatment of THP-1 cells with GW843682X for 12 to 24 h induced significant cell death. However, this cell death did not contribute to the down-regulation of TNF-α induction. TNF-α gene expression began at 1 h after Pam3CSK4 or LPS treatment, and protein expression began at 6 h after Pam3CSK4 or LPS treatment (data not shown), which preceded the induction of cell death (12 h). This conclusion was further confirmed by the observation that PLK1 inhibition selectively down-regulated the induction of TNF-α by Pam3CSK4 and the induction of TNF-α and IL-8 by LPS, but not all cytokines induced by TLR activation, PLK1 inhibition selectively inhibited ERK and NF-κB signal transduction, but not all TLR-elicited signal transduction. 

TLRs and their adaptor proteins have been reported to be down-regulated for the regulation of a ligand-induced response [23-26]. To test whether GW843682X-induced inhibition of the Pam3CSK4- and LPS-mediated responses is dependent on the down-regulation of TLRs or their signal adaptor protein, the effect of GW843682X on the expression of TLR2, TLR4, and MyD88 was detected. The results showed that GW843682X did not regulate TLR2 or TLR4, but it decreased the expression of MyD88. However, the down-regulation was observed based on the treatment of GW843682 for 12 to 24 h, which was after the induction of TNF-α. Together with the reasons mentioned above, we concluded that the down-regulation of MyD88 did not contribute to the inhibition of Pam3CSK4- and LPS-induced TNF-α.

Ligand binding to TLRs recruits signal adaptor molecules, including TIRAP and MyD88, to elicit the phosphorylation of MAPKs and NF-κB, which are responsible for the induction of pro-inflammatory cytokines. The block of MAPK and NF-κB signaling down-regulates the inflammatory response induced by TLR activation [27,28]. In THP-1 cells, Pam3CSK4 and LPS induced TNF-α production, which was dependent on the activation of MAPKs and NF-κB. Therefore, we investigated the effect of PLK inhibition on Pam3CSK4- and LPS-induced activation of MAPK and NF-κB, and we found that pre-treatment of THP1 with PLK inhibitor GW843682X down-regulated the phosphorylation of ERK and NF-κB induced by Pam3CSK4, and the phosphorylation of ERK, p38 and NF-κB induced by LPS. These results suggested that the GW843682X down-regulated TLR-induced TNF-α by blocking MAPK and NF-κB signaling.

PLK1 was involved in the production of TNF-α induced by LPS and Pam3csk4. The inhibition of PLK1 by GW843682X down-regulated the production of TNF-α. Meanwhile, the inhibition of PLK1 induced cell apoptosis and down-regulated the expression of MyD88, which is an important adaptor protein for TLR signaling, suggesting that the long time inhibition of PLK1 should down-regulate TNF-α production by decreasing the number of living cells and by decreasing the activation of TLR signaling induced by ligand re-stimulation. 
